# An Approach to Testing Antivandal Composite Materials as a Function of Their Thickness and Striker Shape—A Case Study

**DOI:** 10.3390/polym16050591

**Published:** 2024-02-21

**Authors:** Emilia Irzmańska, Kamila Mizera, Natalia Litwicka, Kamila Sałasińska

**Affiliations:** 1 Department of Personal Protective Equipment, Central Institute of Labour Protection—National Research Institute (CIOP-PIB), Czerniakowska 16, 00-701 Warsaw, Poland; nalit@ciop.lodz.pl; 2 Department of Chemical, Biological and Aerosol Hazards, Central Institute of Labour Protection—National Research Institute (CIOP-PIB), Czerniakowska 16, 00-701 Warsaw, Poland; kamiz@ciop.pl; 3 Faculty of Materials Science and Engineering, Warsaw University of Technology, Wołoska 141, 02-507 Warsaw, Poland; kamila.salasinska@pw.edu.pl

**Keywords:** impact strength, material thickness, statistical analysis

## Abstract

Our research material comprised antivandal fire-retardant hybrid composites modified with inorganic and organic fillers intended for application in public transport vehicles. This paper presents an approach to studying their impact strength as a function of the composite thickness (3 to 6 mm) and striker shape (hemispherical, semicylindrical, wedge-shaped) used in the experimental stand. Group A composites, made of single fabric layers (*n* = 5), were thinner and their impact strength was lower by 73% than that for Group B composites made of double fabric layers. Study results show an almost threefold improvement in impact strength for a thickness increase of as little as 0.3 mm. Statistical analysis (the Shapiro–Wilk test, *p* > 0.05) did not show any significant differences in the quantitative evaluation of changes (*n* = 3) on the surface of the examined materials caused by impacts with strikers of different shapes. In turn, a linear correlation (Shapiro-Wilk test, *W* = 0.0857, *p* = 0.022) was found between impact strength and the thickness of the studied materials. It was observed that appropriate arrangement of fabrics and powder fillers can lead to a different distribution of forces and energy absorbed by the tested material. A lower impact strength was observed for the composite which had powder fillers in its composition, which caused the formation of microvoids in the structure of the material and thus led to a weakening in their strength properties. An effect of the placement of the glass fabric layer in the composite on the results was also observed. Moreover, SEM evaluation of the composites revealed their layered structure and the impregnation of woven fabrics with resin.

## 1. Introduction

In the context of the public transport system, vandalism is primarily considered the willful destruction of property through setting fire to elements of vehicles or damaging them with sharp or blunt objects [1]. Vandal behaviors are widespread throughout European countries. One of the factors characterizing an act of vandalism is brute force, whose magnitude directly translates into the scale of damage. Thus, in developing composite materials for public transit, one should take into account not only their functional properties, but also their durability in terms of exposure to external factors, such as flames and sharp and blunt objects, as well as in terms of their ability to withstand, e.g., the vertical forces acting on passenger compartment elements [2].

Frequently occurring acts of vandalism involve impacts leading to irreversible changes in material structure. Damage mechanics, which is a branch of science concerned with the description of impact phenomena, defines changes initiated in material structure in response to external loads, as well as their consequences. Understanding the mechanism of vertical forces acting on an object makes it possible to determine the degree of structural degradation resulting from an impact. For this reason, when developing new materials, it is necessary to ensure a substantial level of integrity in all composite layers, as well as a high mechanical strength and load-bearing capacity. Conversely, as a result of an impact, an inadequate material structure may lead to matrix or fiber breaks as well as delamination [3]. Impact-induced damage to composite laminates can be thus classified into “intralaminar” (comprising fiber fractures, matrix cracking or plasticity, fiber-matrix debonding) and “interlaminar” [4].

Technological advancement entails increasing the requirements for materials used in vehicles to ensure appropriate structural strength properties. Unlike traditional materials, such as steel or aluminum, polymer composites have the advantage of a lower density combined with higher rigidity and hardness, as well as improved anti-corrosion properties [5,6]. In turn, reducing the weight of an element by 10% translates into lower vehicle loading and an approx. 6–8% decrease in fuel consumption. Considering the availability and prices of fossil fuels, the specific weight of structures is one of the key factors in an industry like transportation [7]. One solution to the problem of heavy structural elements and equipment in buses is the use of aluminum (27% weight reduction). However, the issue of material thickness persists. Replacing aluminum materials with composites containing glass fibers enables reduced composite thickness without compromising strength properties as compared to heavy metals [8]. A possible solution is to develop hybrid composites consisting of a matrix with reinforcement in the form of fibers or organic or inorganic powder fillers [9]. Glass, carbon, and aramid fibers, as well as mineral and organic particles (including basalt, flax, or hemp), are often used as fillers for polymer composites, primarily to enhance their mechanical properties [10,11]. The type of reinforcement determines the properties of the composite, with the steps of modeling components and their adhesion being crucial [12]. Additionally, when optimizing hybrid materials, one should consider cost minimization and pollution reduction.

An important stage in developing composite prototypes is assessing the functionalization of additives and composite structures in terms of their potential applications and purpose [13,14]. To that end, our research team [15] developed and validated research procedures that included the evaluation of parameters such as cut, flame, and impact strength [16]. Research on assessing hybrid composites that are highly resistant to acts of vandalism has expanded the scope of analysis to encompass the impact of vertical force on composite materials varying in composition and thickness. This paper presents an approach involving the adaptation of research methodology to the evaluation of composite materials in terms of their impact strength against different striker shapes, taking into account material thickness. A flexible approach to testing the impact strength of composites will make it possible to gain knowledge about the effects of their thickness on their mechanical parameters, which is particularly important in the case of public transport applications where durable and lightweight materials must be used. This approach may also serve as a new way of researching the impact strength of composite materials challenged with variously shaped tools.

## 2. Materials and Methods

### 2.1. Experimental Composite Materials

Examples of experimental materials are antivandal fire-retardant hybrid composites modified with inorganic and organic fillers. Composite samples were fabricated for the needs of the present study in the Central Institute for Labour Protection—National Research Institute (CIOP-PIB) laboratory under repeatable and reproducible conditions. They are uniform in static terms and can be used in reliable assessment of the applied test. The fabricated experimental materials are subject to patent P.442733.

The following fabrics were used for the preparation of the composites: a Biax 400 g/m^2^ carbon fabric from Saertex, (Saerbeck, Germany), which is a sewn two-way (+45/−45°) fabric with a surface density of 410 g/m^2^; an aramid fabric from P.P.H.U. Surfpol (Rawa Mazowiecka, Poland), which is a linen weave fabric (twill 2/2) with a surface density of 110 g/m^2^ made from a 42 tex yarn; a Biax 400 g/m^2^ glass fabric from Saertex (Saerbeck, Germany) and a 200 g/m^2^ Interglass, which is a two-way (+45/−45°) fabric with a surface area of 411 g/m^2^ made from E-glass from Milar (Grodzisk Mazowiecki, Poland); Basfiber^®^ BT11/1 from Stone Vek (Moscow, Russia), which is a 380 ± 25 g/m^2^ twill fabric; a linen fabric from Safilin (Sailly-Sur-La-Lys, France), which is a 500 g/m^2^ twill fabric made from a 400 tex yarn.

The powder fillers used were: superfine raw vermiculite with grain size in the range of 0.3–1 mm (at least 80%) from Vermeko Sp. z.o.o., (Lublin, Poland). The bulk density of the raw mineral material was 110–130 kg/m^3^ with the admixture of other rocks not exceeding 10%; CO_2_-filled glass beads with a diameter of 30–115 µm and a bulk density of approx. 120 g/L, with the trade name Mikrobalon DT-99 delivered by PROGMAR (Leszno, Poland); hazelnut shells obtained from the company AGRO Jarosław Seroczyński (Nadbrzeż, Poland). Preparatory procedures included drying and grinding the raw material using a MUKF-10 laboratory sieve mill from Młynpol P.P.H (Chwaszczyno, Poland). During grinding, a sieve with a mesh size of 0.2 mm with teardrop shape was used.

Hybrid composites were prepared using the popular vacuum bag technique. RenLam LY 113 epoxy resin and Ren HY 97-1 hardener, both from Huntsman Advances Materials GmbH (Basel, Switzerland) were mixed using a proLAB 075 stirrer from GlobimiX Ltd. (Ząbkowice Śląskie, Poland) at a 100:30 wt. ratio. The resin was evenly spread on each of the pre-cut fabric layers lying on parallel horizontal sheets of polyethylene and Teflon film using spatulas, brushes, and laminating rollers. In the case of composites with powder filler, they were added to epoxy resin using specific fabrics, respectively:

Vermiculite to the aramid fabric layer, glass beads to or instead of the glass fabric layer, hazelnut shells to the linen fabric layer (Group A composites);

- Vermiculite instead of one aramid fabric layer, hazelnut shells instead of one linen fabric layer (Group B composites).

The order of the fabrics was denoted with the letters a–e in Table 1 and Table 2, except for composite W5o (Table 1) consisting of six glass fabric layers, which served as a reference material.

Table 1 and Table 2 contain photographs of the external surface of the composites acquired using a stereoscopic microscope (Opta-Tech, Warsaw, Poland) at a magnification of 8×. Table 1 and Table 2 present the composition of the tested hybrid composites. Variation in the composition of the presented composites is intended to enable the examination of the influence of individual layers on the mechanical and strength properties of the tested materials. Finally, tests were conducted and results presented for 10 composites of different composition and thickness.

### 2.2. Thickness Measurement

Prior to tests, the thickness of composite materials was taken in quintuplicate using a dial thickness gauge (Instom, Piastów, Poland) at a pressure of 22 kPa. Thickness was measured in the middle of the sample, which was in direct contact with elements of the measurement system during impact strength tests.

### 2.3. Microstructural Analysis

Composite structure was examined using a TM 3000 scanning electron microscope (Hitachi, Tokyo, Japan). In order to improve conductivity, the materials were sputter-coated using a Polaron SC7640 device. The materials were examined at a magnification of 100× and an accelerating voltage of 5–15 kV.

### 2.4. Impact Strength

Impact strength was measured using a universal tester (Pegasil, Portugal). The reference standard was PN-EN ISO 20344:2022-04 for measuring the impact strength of toe caps used in safety and protective footwear [18,19]. Due to the different purpose of the methods contained in the aforementioned standard, the experimental stand was modified to enable testing flat materials of different thicknesses with the characteristics described in this study. Following this approach, we were able to test all fabricated composite materials irrespective of whether or not they were consistent with sample holder dimensions.

Since the tested materials varied in thickness, the experimental stand elements had to be modified to expand the range of impact strength test options [20]. This approach made it possible to determine the impact strength parameter for all the fabricated composites, whether or not their thickness corresponded to the clearance of the sample holder.

The measurement procedure was the same for both thinner and thicker composites, but in the case of Group A of materials, a silicone insert was used to make sure the striker properly impacted the central part of the sample. Due to its physicochemical properties, the insert material increased composite friction in the holder, which helped to immobilize the sample upon striker contact.

**Figure 1:**
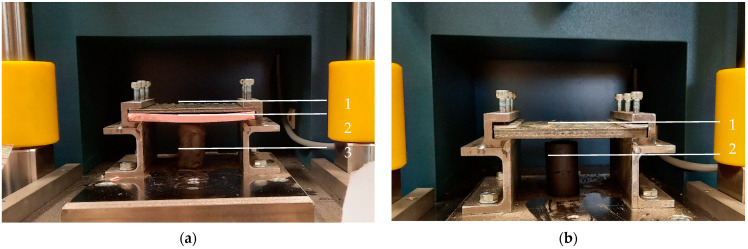
Holder for mounting antivandal composite materials; (**a**) setup for testing thin composites (group 1): 1—sample; 2—silicone insert; 3—oven-bake modeling clay cylinder; (**b**) setup for testing thick composites (group 2): 1—sample; 2—oven-bake modeling clay.

Composite samples with the dimensions 100 × 100 mm were placed one by one in the holder shown in Figure 1a,b, at a distance of 50 cm from a 2 kg striker. Impact simulations were performed in triplicate, using strikers of three shapes: (1) hemispherical; (2) semicylindrical; and (3) wedge, as shown in Table 3. For the purpose of the study, we prepared cylinders of oven-bake modeling clay with a diameter of 25 mm and a height of 40 mm, which were placed underneath the central part of the sample. After positioning all elements of the experimental system, the striker was dropped with the impact energy being equal to 100 J.

The study aimed to evaluate the impact strength of composite materials both qualitatively and quantitatively. All deformations of the studied materials were assessed organoleptically. Quantitative evaluation was performed by measuring the depression of the oven-bake modeling clay cylinder placed under the sample, in its central part (the cylinder was depressed as a result of sample deformation upon impact). Thus, the height of the depressed modeling clay cylinder was used for estimating the degree of composite damage [15].

### 2.5. Statistical Analysis

Statistica 13 software package was used to determine basic statistics and test research hypotheses. The Shapiro–Wilk normality test for both groups of composite materials was conducted on the basis of descriptive statistics. One-way analysis of variance (ANOVA) was used to evaluate impact strength depending on striker shape (number of clustering variable categories *n* = 3). The equality of variances was tested by means of the Levene test (for the same sample sizes). The obtained results were deemed statistically significant in the range of *p* < 0.05 for alpha = 5%. In addition, the one-way relationship between the two variables was determined using the non-parametric Spearman rank correlation test.

## 3. Results

### 3.1. Thickness Measurement

The study material consisted of Group A—with a single fabric layer—and Group B—with double fabric layers of composite materials produced by vacuum bag lamination.

After measuring the thickness of the resulting composite materials, we identified two groups of composites characterized by different thickness ranges:

- Group A—with a single fabric layer of materials with a thickness ranging from 3.3 to 4.1 mm (W1o–W5o);
- Group B—with double fabric layers of materials with a thickness ranging from 4.4 to 6.3 mm (W1a–W5a).

Thickness measurement results for both groups of fabricated materials are given in Table 4. Differences resulting from the thickness of the tested composites are related to the number of fabric layers and powder fillers. The materials were also classified in terms of differences in their composition.

**Figure 2:**
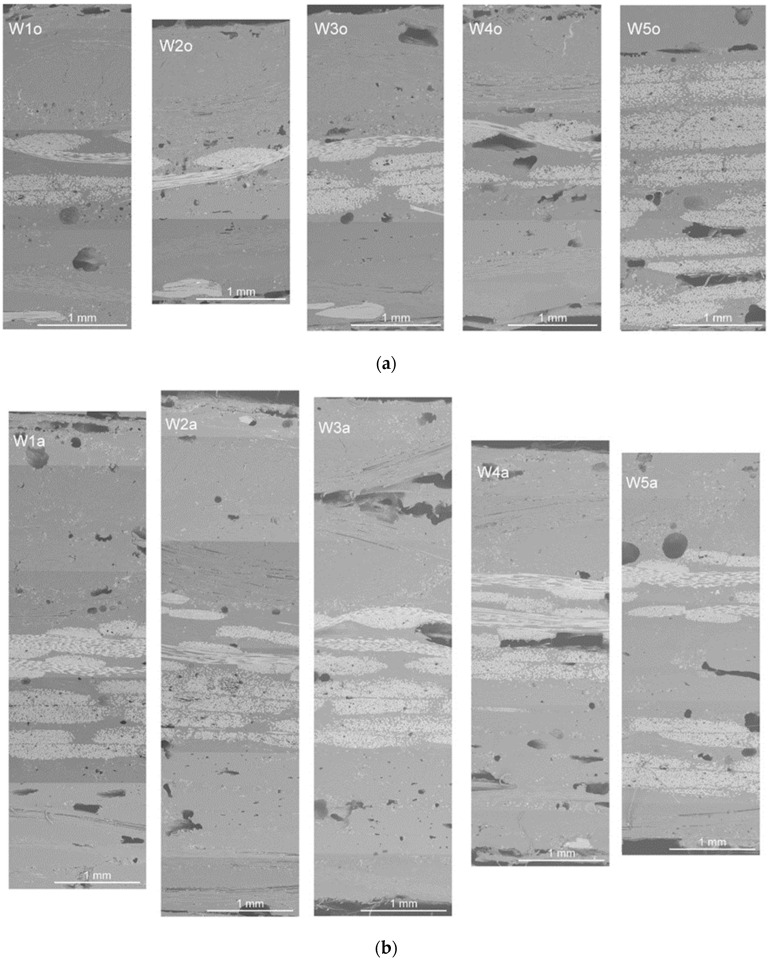
Cross-sections of hybrid composites from Group A—with single fabric layers (**a**)—and Group B—with double fabric layers (**b**)—according to the composition of materials presented in Table 1 and Table 2, respectively.

The materials’ microstructures were examined using a scanning electron microscope (SEM) with the obtained cross-section images shown in Figure 2. The acquired SEM images of composites revealed that, although fibers penetrated through the resin, numerous voids of different sizes and morphology were observed. The existence of voids between and within the layers is caused by the poor saturation of the matrix. In turn, their presence in the outer layer may result from the excessively long use of vacuum [17]. Differences in the appearance and directionality of fabrics made it possible to separate out the various layers. The images show bundles of inorganic fibers from sewn woven fabrics (glass, basalt, carbon) and inorganic powder fillers and additives (vermiculite, glass beads, fire retardants), but it is much more difficult to identify organic fibers and fillers (aramid, flax, nut shells). The layered structure is best visible for the sample W5o, which is a reference sample consisting of six six layers of fiberglass woven fabric.

### 3.2. Impact Strength Parameter

**Figure 3:**
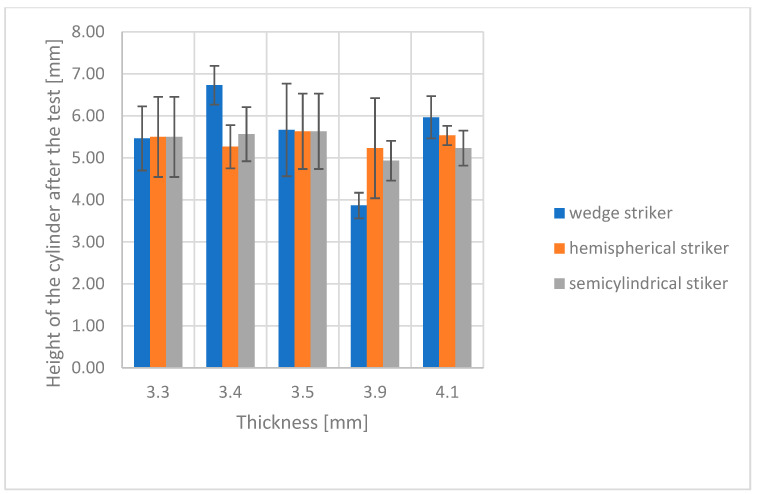
Impact strength of Group A—with a single fabric layer.

**Figure 4:**
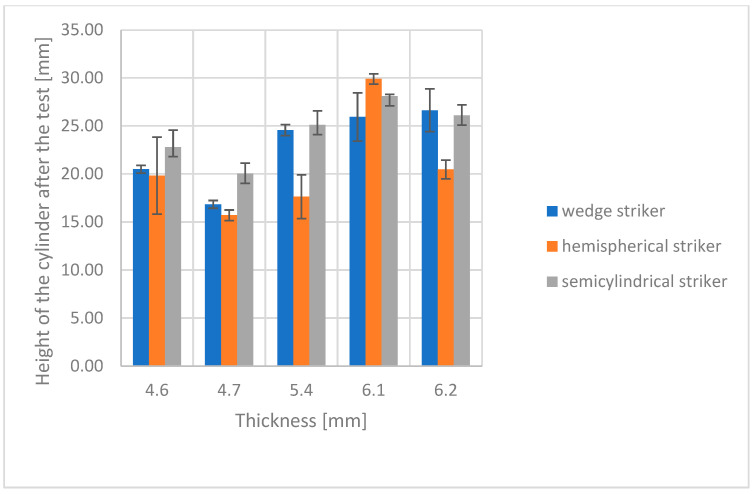
Impact strength of Group B—with double fabric layers.

Impact strength results for both groups of composites are shown in Figure 3 and Figure 4. The diagrams present the relationship between modeling clay cylinder height after impact by strikers of different shapes and the thickness of the studied composite.

**Figure 5:**
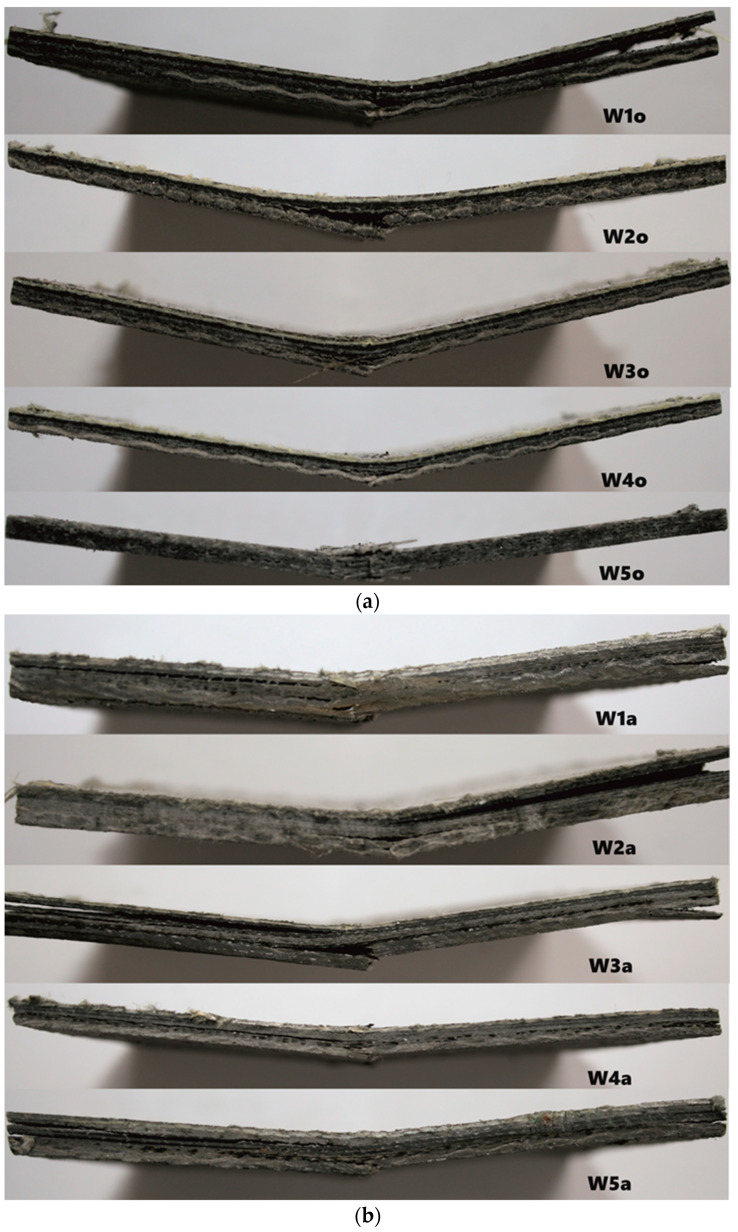
Images of post-destruction hybrid composites from Group A—with a single fabric layer (**a**)—and B—with double fabric layers (**b**).

Visual analysis of the diagrams indicates substantial differences in modeling clay cylinder height following impact for thicker antivandal composite materials (Group B), but not for the thinner ones (Group A). These differences are attributable to different energy absorption patterns upon impact. The thicker the composite, and thus the more fabric layers there are, the greater the proportion of the impact energy absorbed by the surface layers of the composite. The distribution pattern of impact energy also depends on the type of fabrics applied [21]. Error bars in the diagrams indicate standard deviations for three replicates. In all cases, the determined coefficient of variation was within a 5% range. A detailed statistical analysis is given in Section 3.3. Representative images of post-damage samples are shown in Figure 5. The observed differences in the nature of damage depend on the extent of resin penetration in the various fabric layers. The delamination of composites is also affected by the use and type of powder fillers. Their addition to the matrix may give rise to microvoids, which could compromise the strength of the resulting material [22]. This is attributable to the poor adhesion of the reinforcement to the matrix. Also of the essence is the chemical bonding of the matrix to the surface of the applied fillers [23]. In the literature, there are some publications reporting a lower strength of composites containing vermiculite [24]. On the other hand, the application of glass fillers with coating ensures good adhesion to the matrix, resulting in good mechanical properties [25].

For Group A composites, the largest differences in resistance and impact strength were observed for the wedge striker. The lowest impact strength was observed for the W3o composite. This is due to the presence of powder fillers in its composition, which cause the formation of hollow micro spaces in the structure of the material, and thus lead to a weakening of their strength properties. Organic fillers exhibit high absorption, which may cause lower adhesion between the reinforcement and the matrix. In the case of W2o and W5o composites, the same impact strength values are observed regardless of the shape of the striker. For the W2o composite, the SEM images (Figure 2a) showed very good adhesion between the fabrics, powder fillers, and resin. However, in the case of the W-5o composite, the main reinforcement is glass fabric, which is known for its strength properties, especially in the case of tensile strength [26]. For Group B composites, large discrepancies are observed in the values of rubber roller heights. The resistance to impact with tuples is much higher than for thinner composites (Group A). The highest resistance was characterized by composite W2a, whose reinforcement was thinner by one layer of carbon fabric. In contrast, the lowest resistance was observed for composite W4a, which had a single layer of glass fabric. Appropriate arrangement of fabrics and powder fillers can lead to a different distribution of forces and energy absorbed by the tested material [5].

### 3.3. Impact Strength Parameter

**Figure 6:**
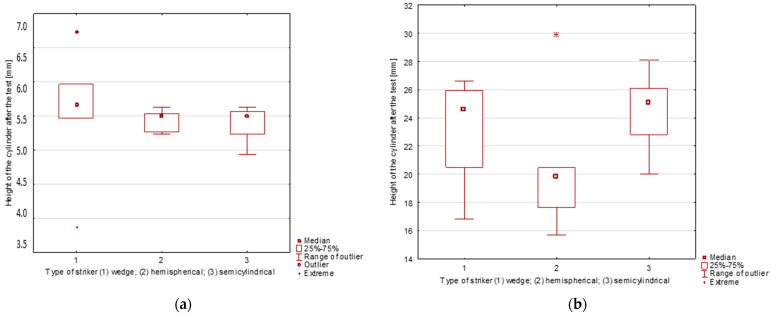
Box-and-whisker diagram (**a**) for the Group A antivandal composite materials and (**b**) for the Group B antivandal composite materials.

The basic statistics determined from mean modeling clay cylinder height after impact (measured in triplicate) for the two measurement groups, taking into account striker shape, are given in Table 5. The Shapiro–Wilk test revealed that the assumption of normal distribution was met for each clustering variable category (*p* > 0.05). In order to visualize the results, the basic statistical parameters are presented in a box-and-whisker plot, are given in Figure 6.

The Group A antivandal composite materials, characterized by a lower thickness, exhibited a smaller dispersion of results as compared to the second group of (thicker) composites, as evidenced by lower kurtosis and a higher degree of clustering around the mean. In the first case, the distribution is left-tailed (skewed towards lower values), while in the second case, it is right-tailed. In both cases there are extreme values, but outliers were found in the Group A antivandal composites for wedge striker impacts (the presence of both extreme and outlying observations was confirmed using the Grubbs test). Due to the fact that we compared results for all composite materials with a view to evaluating the impact of strikers with different shapes, no observations were rejected, and the statistical analysis involved the real data set.

Having conducted tests in five replicates for each striker shape, with the results characterized by a normal distribution, the research hypothesis was evaluated using parametric tests. Further analysis was conducted to verify the following hypotheses:

H_0_: The impact strength results obtained with the use of strikers with different shapes are statistically significant for a given group of materials;

H_1_: The impact strength results obtained with the use of strikers with different shapes are not statistically significant for a given group of materials.

One way ANOVA results for striker shape as a clustering variable is given in Table 6 for the two groups of antivandal composite materials.

Statistical analysis shows the null hypothesis to be true for both groups of materials. Thus, striker shape does not lead to statistically significant differences in post-impact modeling clay cylinder height, which is defined as the impact strength parameter.

An additional relationship evaluated in this study was the correlation between the mean post-impact modeling clay cylinder height and the thickness of all composites. Analysis involved results for both composite material groups. The dependent variable was defined as post-impact modeling clay cylinder height, while the independent variable was the thickness of all composite materials. Prior to correlation evaluation, data distribution was found to be not normal using the Shapiro–Wilk test (*W* = 0.0857 and *p* = 0.022). Consequently, further analysis was conducted using the non-parametric Spearman rank correlation test, with the results given in Table 7.

The obtained coefficients of correlation, *r*, between the dependent and independent variables are within the range <−1, 1>, with their values being close to one which indicates a very strong linear correlation between the studied parameters.

## 4. Conclusions

Our modification of an experimental stand for impact strength measurement made it possible to test composite materials with various characteristics differing in thickness. The applied two-pronged approach showed that impact strength is correlated with the thickness of composite materials. Statistical analysis revealed no significant differences in resistance to impacts by strikers with different shapes.

However, considerable differences in the energy absorption patterns were found for the thicker composites. The obtained results for impact strength were also affected by the type of fabrics and powder fillers used in the external layers of the composite. The composite with the greatest impact strength in Group B was W2a, which had one carbon fabric layer fewer than the other composites, while the lowest impact strength was recorded for the composite with one glass fabric layer fewer than the others. Similarly, in Group A, a high impact resistance was found for composite W5o, which consisted solely of glass fabric layers.

These results indicate that the presence and amount of glass reinforcement had a significant effect on the impact strength of the fabricated composites.

The presented flexible approach to examining the impact strength of composite materials will enable the expansion of knowledge on the effects of thickness on the mechanical parameters of materials, and especially in terms of the materials used in public transit vehicles, which must be characterized by a low weight and good strength parameters.

## Figures and Tables

**Table 1:** Composite materials: Group A—with single fabric layers (*n* = 5) [17].

Sample Symbol	W1o	W2o	W3o	W4o	W5o
Photograph	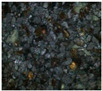	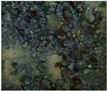	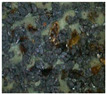	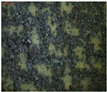	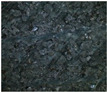
aramid fabric	1a	1a	1a	1a	
carbon fabric	1b	1b	1b	1b	
glass fabric	1c		1c	1c	6c
basalt fabric	1d	1d	1d	1d	
linen fabric	1e	1e	1e	1e	
vermiculite	1a	1a	1a		
glass beads	1c	1c			
nut shells	1e	1e	1e		

Note: 1 and 6 represent the number of layers, while a–e represent the order of layers.

**Table 2:** Composite materials: Group B—with double fabric layers (*n* = 5) [17].

Sample Symbol	W1a	W2a	W3a	W4a	W5a
Photograph	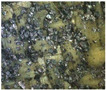	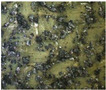	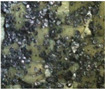	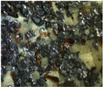	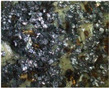
aramid fabric	2a	2a	1a	2a	2a
carbon fabric	2b	1b	2b	2b	2c
glass fabric	2c	2c	2c	1c	2b
basalt fabric	2d	2d	2d	2d	2d
linen fabric	2e	2e	2e	2e	2e
vermiculite					1a
nut shells					1e

Note: 1 and 2 represent the number of layers, while a–e represent the order of layers.

**Table 3:** Striker shapes: (1) wedge; (2) semicylinder; (3) hemisphere.

Image	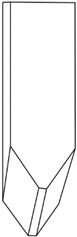	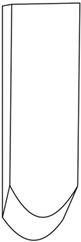	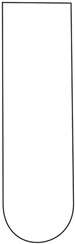
Dimensions	radius (R) = approx. 25 mm	radius (R) = approx. 10 mm	angle = 90° and radius (R) = approx. 3 mm
Symbol	(1)	(2)	(3)

**Table 4:** Composite materials: Group A—with a single fabric layer (*n* = 5); Group B—with double fabric layers (*n* = 5).

**Sample Symbol** **Group A—with Single Fabric Layer**	**W1o**	**W2o**	**W3o**	**W4o**	**W5o**
Thickness [mm]	4.1	3.3	3.9	3.4	3.5
Standard deviation	0.2	0.1	0.2	0.1	0.2
**Sample symbol** **Group B—with double fabric layers**	**W1a**	**W2a**	**W3a**	**W4a**	**W5a**
Thickness [mm]	5.4	6.1	6.2	4.7	4.6
Standard deviation	0.3	0.2	0.2	0.1	0.1

**Table 5:** Basic statistics for mean post-impact modeling clay cylinder height as a function of striker shape.

Number	Striker Type	Mean	Median	Min	Max	Variance	Standard Deviation	Skewness	Kurtosis	W	*p*
A	1	5.54	5.67	3.87	6.73	1.11	1.05	−1.04	2.10	0.927	0.576
	2	5.43	5.50	5.23	5.63	0.03	0.17	−0.26	−2.52	0.892	0.368
	3	5.37	5.50	4.93	5.63	0.08	0.29	−1.05	−0.11	0.891	0.362
B	1	22.89	24.57	16.83	26.63	9.80	17.12	4.14	−0.89	−0.88	0.894
	2	20.71	19.83	15.70	29.90	14.20	29.96	5.47	1.58	2.95	0.855
	3	24.43	25.10	20.03	28.10	8.07	9.67	3.11	−0.48	−0.39	0.980

Legenda: Composite materials: Group A—with a single fabric layer (*n* = 5) (Figure 6a); Group B—with double fabric layers (*n* = 5) (Figure 6b); 1—wedge striker; 2—semicylindrical striker; 3—hemispherical striker; W–Shapiro–Wilk coefficient; *p*—normal distribution at *p* > 0.05.

**Table 6:** Analysis of variance between means from trials for striker shape as a clustering variable.

Group of Materials	Levene Test		ANOVA Test	
	*F*	*p*	*F*	*p*
1	2.563	0.118	0.088	0.917
2	0.361	0.704	0.923	0.424

**Table 7:** Correlation between impact strength and antivandal composite material thickness (*n* = 30).

	Dependent Variable	Independent Variable
Dependent variable	1	0.813
Independent variable	0.813	1

## Data Availability

Data are contained within the article.
